# The hepatic ectonucleotide pyrophosphatase/phosphodiesterase 1 gene mRNA abundance is reduced by insulin and induced by dexamethasone

**DOI:** 10.1590/1414-431X20176980

**Published:** 2018-03-01

**Authors:** Huiwen Ma, Ping Wang, Dan Jin, Ting Jia, Hong Mao, Jiandi Zhang, Shi Zhao

**Affiliations:** 1 Department of Endocrinology, Wuhan Central Hospital, Wuhan, Hubei, China; 2 Yantai Center for Animal Disease Control, Yantai, Shandong, China; 3 Department of Anesthesiology, Shandong Provincial Hospital, Jinan, Shandong, China; 4 Department of Laboratory Medicine, Hubei University of Chinese Medicine, Wuhan, Hubei, China; 5 Yantai Zestern Biotechnique Co. Ltd., Yantai, Shandong, China

**Keywords:** Ectonucleotide pyrophosphatase/phosphodiesterase 1, Insulin, Dexamethasone, Hepatocytes, Regulation of gene expression

## Abstract

Hormones regulate hepatic gene expressions to maintain metabolic homeostasis. Ectonucleotide pyrophosphatase/phosphodiesterase 1 has been thought to interfere with insulin signaling. To determine its potential role in the regulation of metabolism, we analyzed its gene (*Enpp1*) expression in the liver of rats experiencing fasting and refeeding cycles, and in primary rat hepatocytes and human hepatoma HepG2 cells treated with insulin and dexamethasone using northern blot and real-time PCR techniques. Hepatic *Enpp1* expression was induced by fasting and reduced by refeeding in the rat liver. In primary rat hepatocytes and HepG2 hepatoma cells, insulin reduced *Enpp1* mRNA abundance, whereas dexamethasone induced it. Dexamethasone disrupted the insulin-reduced *Enpp1* expression in primary hepatocytes. This is in contrast to the responses of the expression of the cytosolic form of phosphoenolpyruvate carboxykinase gene to the same hormones, where insulin reduced it significantly in the process. In addition, the dexamethasone-induced *Enpp1* gene expression was attenuated in the presence of 8-Br-cAMP. In conclusion, we demonstrated for the first time that hepatic *Enpp1* is regulated in the cycle of fasting and refeeding, a process that might be attributed to insulin-reduced *Enpp1* expression. This insulin-reduced *Enpp1* expression might play a role in the development of complications in diabetic patients.

## Introduction

Rapid global economic development and improvement of living standards is associated with the increasing incidence of obesity and other human metabolic diseases, such as type 2 diabetes (1), which has become a public health concern. Human obesity and type 2 diabetes are associated with profound changes of hepatic lipid and glucose metabolism, attributed partially to the altered expression of hepatic genes (2). Insulin is essential for the control of blood glucose homeostasis, partly through the regulation of gene expressions in a variety of tissues and organs.

It has been shown that insulin-regulated gene expression contributes to glycolysis, glycogenesis, lipogenesis (fatty acid biosynthesis), and gluconeogenesis (glucose production) in the liver. For instance, insulin increases the hepatic expression of glucokinase gene (*Gck*) (3), the first enzyme for hepatic glycolysis. On the other hand, insulin reduces the expression of the cytosolic form of phosphoenolpyruvate carboxykinase gene (*Pck1*) (4), the rate limiting enzyme for gluconeogenesis. In addition, the hepatic expression of *Pck1* gene can be induced by glucocorticoids and activation of protein kinase A pathway (4). The development of insulin resistance is associated with the disruption of insulin-regulated hepatic gene expression (5). Therefore, understanding the mechanism of insulin resistance is urgent.

One protein that has been implicated to cause insulin resistance is ectonucleotide pyrophosphatase/phosphodiesterase 1 (ENPP1) or plasma cell membrane glycoprotein 1, which is thought to hydrolyze ATP to generate inorganic pyrophosphate (PPi) plus AMP or inorganic phosphate (Pi) plus ADP (6). ENPP1 is a member of nucleotide pyrophosphatases/phosphodiesterases family with 5 members, and it can exist as a soluble form (7). Additionally, it has been shown that ENPP1 isolated from the mouse liver also catalyzes the hydrolysis of 2′3′-cGAMP (8). The main physiological function of ENPP1 has been related to bone mineralization, as *Enpp1−/−* mice demonstrate defects in bone mineralization process (9,10). Mutations within this gene lead to artery calcification (11). Another member of the family, ENPP2, also known as autotaxin, has been considered a secreted lysophospholipase D that is produced in adipocytes and is responsible for the synthesis of lysophosphatidic acid. ENPP2 has been implied to affect insulin sensitivity and induce development of obesity (12).

Originally, ENPP1 was identified as an antigen to analyze the specificity of polyclonal allo-antisera raised in DBA/2 mice against the BALB/c myeloma cell line MOPC-70A (13). In that study, positive interactions of the antiserum with antigens in the homogenates of liver, brain, spleen, kidney, and lymph node cells were observed (13). An immunohistochemistry study also showed that ENPP1 can be detected in these tissues (14). Genetic variations and polymorphisms of ENPP1 have been associated with insulin resistance in human subjects (15,16). However, a study including 8,089 UK Caucasians with type 2 diabetes and obesity did not find association between variants of ENPP1 and these diseases (17).

On the other hand, experimental results have indicated that ENPP1 might contribute to insulin resistance. Transgenic mice over-expressing the human ENPP1 driven by the CMV promoter had hyperglycemia and hyperinsulinemia with impaired glucose tolerance test, and reduced glucose uptake in the muscle (18). Elevated ENPP1 protein level and alkaline phosphodiesterase I activity were reported in liver, muscle, and brain, but not adipose tissue (18). When recombinant adenovirus was used to express human ENPP1, its over-expression in the liver caused the impairment of glucose tolerance test, reduction of insulin signaling, and elevation of mRNA abundance levels of gluconeogenic genes (19). The mechanism of ENPP1 action has been attributed to its interaction with the insulin receptor α subunit in MCF-7 cells (20). However, this view was challenged as in NIH-3T3 fibroblasts with stable overexpression of insulin receptor, neither wild-type ENPP1 nor the K173Q mutant in transiently transfected condition has the ability to interact with insulin receptor (21). Moreover, the over-expression of ENPP1 did not change the insulin receptor autophosphorylation status shown in immunoblot (21).

Given the fact that ENPP1 is detected in the liver and its hepatic over-expression leads to insulin resistance, we aimed to investigate the regulation of hepatic *Enpp1* expression.

## Material and Methods

### General reagents

Various cell culture reagents, including DMEM medium, M199 medium, sodium penicillin, streptomycin sulfate, fetal bovine serum, MEM medium, and RNase-free DNase were purchased from Thermo Fisher Scientific (USA). Liver perfusion buffer and liver digestion buffer were obtained from Invitrogen, currently Thermo Fisher Scientific. Dexamethasone, 8-bromoadenosine 3′,5′-cyclic monophosphate (8-Br-cAMP, cell permeable cAMP analog), 3,3′,5-tri-iodothyronine (T3), insulin, RNA loading buffer, and formamide were purchased from Sigma (USA). Reagents for cDNA synthesis and 2× SYBR Green PCR Master Mix were obtained from Applied Biosystems (USA). RNA STAT 60 was from TEL-TEST Inc. (USA). Rat tail collagen I-coated 60-mm dishes were obtained from BD Biosciences Discovery Labware (USA).

### Animal maintenance and studies

Male Sprague-Dawley rats were obtained from the animal laboratory at Tongji Medical College (Wuhan, China) for the fasting and refeeding study, and from Harlan Breeders (USA) for hepatocyte isolation as published previously (22). They were housed in colony cages, maintained on a 12-h light/12-h dark cycle. All animal experiments were performed with the approval of Institutional Animal Care and Research Advisory Committee of respective institutions.

For the fasting and refeeding studies, two studies were conducted. In experiment #1, four Sprague-Dawley rats (200–250 g) were fasted for 36 h. Then, 2 animals received the chow diet for 12 h, and the remaining 2 were fasted during this period. In experiment #2, 15 Sprague-Dawley rats at around 250 g were divided into three groups, ad libitum, fasting and refeeding groups. For fasting and refeeding groups, rats were fasted for 36 h and then the refeeding group received a chow diet for 12 h while the fasting group remained fasted during this period. Rats in ad libitum group had free access to the diet during the experiment period. All experimental animals had free access to water during the experimental period before euthanasia.

### Cell culture

HepG2 cells were maintained in MEM supplemented with 10% fetal bovine serum, 100 units/mL sodium penicillin, and 100 µg/mL streptomycin sulfate before they were set up for experiments.

Rat primary hepatocytes were isolated from non-fasting 250-g Sprague-Dawley male rats by the collagenase method with minor modifications. Animals were anesthetized with halothane, and each liver was perfused *in situ* via the portal vein with 100 mL of Liver Perfusion Medium (Invitrogen, USA). The medium was warmed to 37°C and infused at a rate of about 10 mL/min. The liver was then perfused with liver digest medium containing collagenase (Invitrogen) for roughly 15 min at a flow rate of about 10 mL/min. The liver was removed, the hepatic capsule was stripped, and the dissociated cells were dispersed by shaking, followed by filtration at 4°C through gauze into an equal volume of ice-cold DMEM (Invitrogen) containing 5% (v/v) fetal calf serum, 10 mM Hepes (pH 7.4), 100 units/mL of penicillin, and 100 μg/mL of streptomycin. The cells were pelleted and washed twice at 4°C with the same buffer. Aliquots of 2×10^6^ cells were plated onto 60-mm rat collagen I-coated dishes (BD Biosciences Discovery Labware) in the same DMEM supplemented with 5% (v/v) fetal calf serum, 100 nM tri-iodothyronine, 100 units/mL penicillin, 100 μg/mL streptomycin, and 0.1 mg/mL gentamycin. After incubation for 3 to 4 h, the attached cells were washed once with 4 mL of PBS, and incubated in medium 199 supplemented with 100 nM dexamethasone, 100 nM T3, 100 units/mL penicillin, and 100 μg/mL streptomycin sulfate plus 1 nM insulin for 14–16 h until being used for the indicated experiments. Cell viability, as measured by trypan blue exclusion, was always greater than 85%. After incubation for 16 h, the cells were ready for treatment.

### RNA isolation and DNase treatment

To isolate RNA from cultured cells, culture media were removed from culture dishes by aspiration. For each 60-mm dish, 1 mL of RNA STAT 60 was added and swirled to insure coverage. Dishes were kept at room temperature for at least 10 min. Lysates were transferred into 1.5 mL micro-centrifuge tubes by scraping. After adding 0.2 mL of chloroform, the tube was shaken vigorously for 15 s and kept at room temperature for at least 2 min. Samples were spun at about 16,000 *g* for 20 min in a desktop micro-centrifuge at 4°C. About 0.5 mL of upper phase was transferred into a fresh 1.5 mL micro-centrifuge tube containing 0.5 mL of 100% isopropanol. Total RNA was precipitated by centrifugation at 16,000 *g* for 20 min at 4°C. RNA pellet was washed twice with 1 mL of 70% ethanol and dissolved in 88 µL of nuclease-free water. To remove contaminated DNA, total RNA was treated with 4 units of RNase-free DNase for 30 min at 37°C in a final reaction volume of 100 µL. The buffer condition is 10 mM Tris-HCl pH 7.5, 2.5 mM MgCl_2_ and 0.5 mM CaCl_2_. After DNase digestion, 10 µL of DNase inactivation resin was added, mixed and spun down. The DNA-free RNA was transferred into a fresh microcentrifuge tube and its RNA content was determined by Spectronic Genesys 5 spectrophotometer (Thermo Fisher Scientific). The total liver RNA samples were extracted and pooled together for northern blot analysis. RNA samples were stored at -80°C until used.

### Northern blot analysis

Methods for northern blot have been described previously (23). Briefly, about 20 µg of total RNA was suspended into RNA loading buffer, separated by 1% agarose-formaldehyde gel and transferred onto nylon membrane (Hybond-N+). For preparation of *Enpp1* probe, PCR primers, 5′-GAAAGACCACACTTTTACACTC-3′ (forward) and 5′-TTACAACTGCCTTGTTCCATGCC-3′ (reverse), derived from rat *Enpp1* mRNA sequence (NCBI Reference Sequence: NM_053535.1) were used to amplify rat hepatic cDNA. The resulting amplicon of 211 bp containing *Enpp1* mRNA sequence was inserted into the TA cloning vector pCR2.1 (Thermo Fisher Scientific). The plasmid was cut with EcoRI to release the insert, which was purified on agarose gels. The cDNA probes for detecting apolipoprotein E (*Apoe*), fatty acid synthase (*Fas*), *Gck*, insulin-like growth factor-binding protein (*Igfbp-1*), and *Pck1* genes have been published previously (23). The cDNA probes were labeled with [α-^32^P] dCTP using a random primer labelling kit (GE Healthcare Life Sciences, USA). Hybridization was performed at 65°C in ExpressHyb hybridization solution (BD Bioscience Clontech, USA) according to the manufacturer’s instructions. Membranes were exposed to an X-ray film at either 25°C or -80°C to obtain the bands.

### cDNA synthesis and quantitative real-time PCR

First strand cDNA was synthesized from 2 µg of DNA-free RNA with random hexamer primers using the cDNA synthesis kit in a final volume of 100 µL. The levels of mRNA transcripts were measured by quantitative real-time PCR. The sequences of primer sets used are for rat *Enpp1* 5′-CCAAGTCATCCCAAAGAAGAG-3′ (forward) and 5′-GAAGTCCATGATCGGCACAA-3′ (reverse); for rat 36B4 (acidic ribosomal phosphoprotein, P0) 5′-TTCCCACTGGCTGAAAAGGT-3′ (forward) 5′-CGCAGCCGCAAATGC-3′ (reverse); for human *ENPP1* primer 5′-CGATTTTGCCGATTGAGGATT-3′ (forward) and 5′-AAACTGGTGCTGGGAAAGAAGACA-3′ (reverse); for human 36B4 primer 5′-AACATGCTCAACATCTCCCC-3′ (forward) and 5′-CCGACTCCTCCGACTCTTC-3′ (reverse). Each real-time PCR reaction contains, in a final volume of 14 µL, cDNA from 14 ng of reverse transcribed total RNA, 2.33 pmol forward and reverse primers, and 7 µL of 2× SYBR Green PCR Master Mix (Applied Biosystems). PCR was carried out in 96-well plates using the 7300 Real Time PCR System (Applied Biosystems). All reactions were done in triplicate. The PCR conditions are 50°C for 2 min, 95°C for 10 min, followed by 40 cycles of 95°C for 15 s and 60°C for 1 min. The relative amounts of all transcripts in real-time PCR experiment were calculated using the comparative CT method as described earlier (5) using 36B4 as the invariant control. The contents of 36B4 and *Enpp1* transcripts at time 0 were arbitrarily assigned as 1.

### Statistical analysis

Statistical analysis was performed using the SPSS (version 11.0, SPSS Inc, USA). Data are reported as means±SE. The number of experiments represented the independent treatments of indicated cells in different days. An independent-samples *t*-test was used to compare two conditions. Multiple comparisons were analyzed by one-way ANOVA using least significant different (LSD) when equal variance was assumed, and Games-Howell test was used when equal variance was not assumed. Differences were considered to be statistically significant at P<0.05.

## Results

**Figure 1.**
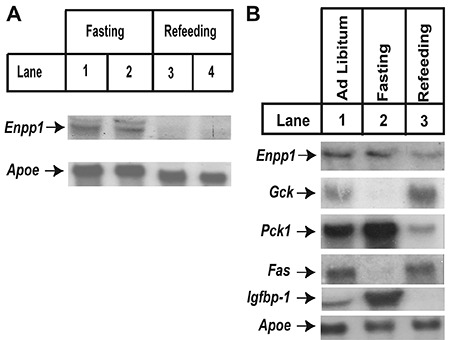
Expression levels of *Enpp1*, *Gck*, *Pck1*, *Fas, Igfbp-1*, and *Apoe* transcripts in the liver of rats in the cycle of fasting and refeeding. *A*, mRNA of *Enpp1* and *Apoe* in the liver of rats (2 in each group) fasted for 48 h or refed for 12 h after a 36-h fasting. *B*, mRNA levels of *Enpp1*, *Gck*, *Pck1*, *Fas, Igfbp-1*, and *Apoe* in the liver of rats (5 rats per group) fed *ad libitum*, fasted for 48 h or refed for 12 h after a 36-h fasting. Total liver RNA pooled from rats in each group was isolated and subjected to northern blot analysis. *Enpp1*: Ectonucleotide pyrophosphatase/phosphodiesterase 1 gene; *Apoe*: apolipoprotein E gene; *Gck*: glucokinase gene; *Pck1*: the cytosolic form of phosphoenolpyruvate carboxykinase gene; *Fas*: fatty acid synthase gene; *Igfbp-1*: insulin-like growth factor-binding protein 1.

The experimental groups are shown in Figure 1A. Compared with fasting state, the hepatic *Enpp1* mRNA level in the refeeding state dropped significantly. As the invariable control gene, the mRNA levels of *Apoe* gene did not change. This observation was further confirmed with other rats under ad libitum, fasting, and refeeding conditions as shown in Figure 1B. The success of the fasting and refeeding procedure was confirmed by examining the expression levels of the cytosolic form of *Pck1*, *Fas*, *Igfbp-1* and *Gck* genes, which are known to be regulated during the cycle of fasting and refeeding (23). Consistent with the results in the literature (4), the mRNA levels of *Pck1* and *Igfbp-1* genes were indeed up-regulated in the fasting state, and they were reduced after refeeding for 12 h. On the other hand, the mRNA levels of *Gck* and *Fas* were reduced in the fasting state and increased significantly after refeeding. As the invariable control, the mRNA levels of *Apoe* gene in ad libitum, fasting, and refeeding states were not different from each other.

**Figure 2.**
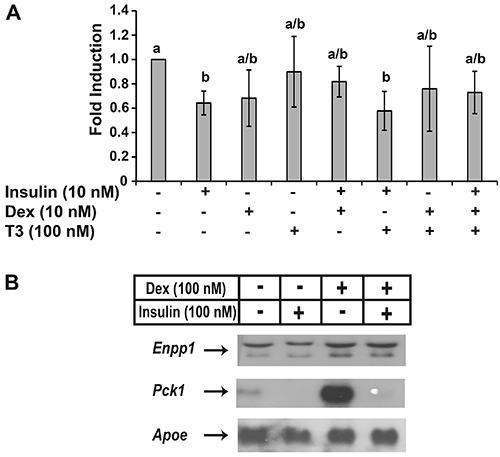
Total RNA was isolated and subjected to real-time PCR analysis (*A*) or northern blot (*B*). *A*, Expression levels of *Enpp1* mRNA in primary hepatocytes treated with 10 nM insulin, 10 nM dexamethasone (Dex), 100 nM T3 or their combinations for 8 h (n=3 independent hepatocyte isolation and treatments). Data are reported as means±SE. P<0.05, different lower-case letters indicate statistical differences (one-way ANOVA). *B*, Expression levels of *Enpp1*, *Pck1*, and *Apoe* mRNA in primary hepatocytes treated with 100 nM insulin, 100 nM Dex or their combination for 16 h. *Enpp1*: ectonucleotide pyrophosphatase/phosphodiesterase 1 gene; *Gck*: glucokinase gene; *Apoe*, apolipoprotein E gene.

Figure 2A shows that the treatments of insulin and insulin combined with T3 for 6 h significantly reduced the *Enpp1* expression whereas T3 alone did not affect its expression. On the other hand, the presence of dexamethasone appeared to disturb the insulin-reduced *Enpp1* expression, suggesting the potential role of dexamethasone on the induction of hepatic *Enpp1* expression. Indeed, the northern blot data shown in Figure 2B demonstrated that the *Enpp1* mRNA level was either slightly reduced or increased in primary hepatocytes treated with 100 nM insulin or 100 nM dexamethasone for 12 h, respectively. As expected, insulin and dexamethasone respectively reduced and induced the *Pck1* expression in the same experiment. The treatment of 100 nM dexamethasone induced *Enpp1* expression even in the presence of 100 nM insulin, while the *Pck1* expression was inhibited under the same treatment. These results demonstrated the dominant role of dexamethasone in the induction of hepatic *Enpp1* expression.

**Figure 3.**
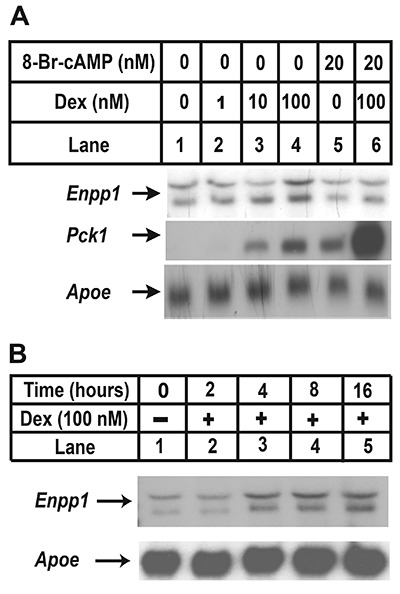
Total RNA was isolated and subjected to northern blot analysis. *A*, mRNA levels of *Enpp1*, *Pck1*, and *Apoe* in hepatocytes treated with 0, 10, 100 nM dexamethasone (Dex) or 20 nM 8-Br-cAMP in the absence or presence of 100 nM Dex for 16 h. *B*, mRNA abundance levels of *Enpp1* and *Apoe* in hepatocytes treated with 100 nM dexamethasone for 0, 2, 4, 8, or 16 h. *Enpp1*: Ectonucleotide pyrophosphatase/phosphodiesterase 1 gene; *Apoe*: apolipoprotein E gene; *Pck1*: cytosolic form of phosphoenolpyruvate carboxykinase gene.

To further examine the role of dexamethasone in the induction of *Enpp1* expression, we measured its expression in primary rat hepatocytes treated with increasing concentrations of dexamethasone (0, 1, 10, and 100 nM) and either 20 nM 8-Br-cAMP or 20 nM 8-Br-cAMP with 100 nM dexamethasone for 6 h. As shown in Figure 3A, dexamethasone at 100 nM, but not 1 and 10 nM, induced the *Enpp1* expression, confirming the data shown in Figure 2A. However, this induction was blunted in the presence of 20 nM 8-Br-cAMP, which itself did not affect the *Enpp1* expression. Figure 3B shows that dexamethasone induced *Enpp1* expression as early as 4 h, and this induction remained for at least 16 h in the current experimental settings.

**Figure 4.**
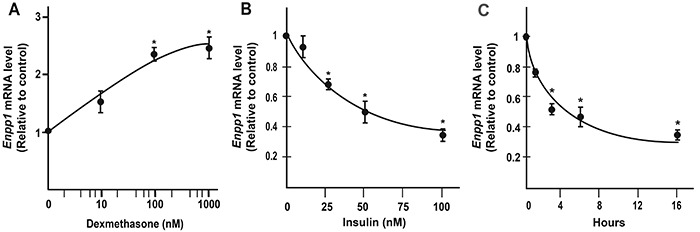
Expression levels of *Enpp1* mRNA in HepG2 cells. *A*, *Enpp1* mRNA in HepG2 cells treated with 10, 100, or 1000 nM dexamethasone for 16 h. *B*, *Enpp1* mRNA levels in HepG2 cells treated with 5, 25, 75, or 100 nM insulin overnight. *C*, *Enpp1* mRNA levels in HepG2 cells treated with 100 nM insulin for 4, 8, 12, and 16 h. Total RNA was isolated and subjected to real-time PCR analysis (n=3 independent treatments). Data are reported as means±SE. *P<0.05, compared to untreated controls for *A* and *B* or time 0 for *C* (Student’s *t-*test).

To determine whether the observed hormonal regulations are not limited to primary rat hepatocytes, we used real-time PCR to examine the effects of insulin and dexamethasone on the *Enpp1* mRNA abundance in HepG2 cells, a human hepatoma cell line. HepG2 cells have been known for their response to insulin treatment, and have been widely used in metabolic studies. In fact, a recent search of HepG2 and insulin yielded more than 1000 papers in PubMed database. Cells were treated with increasing concentrations of dexamethasone (Figure 4A) or insulin (Figure 4B) for 12 h. As shown in Figure 4A, consistent with our observations in rat primary hepatocytes, the *Enpp1* mRNA level in HepG2 cells started to be induced in a dose-dependent manner by dexamethasone at 100 and 1000 nM, but not at 10 nM. In addition, its expression began to be reduced in a dose-dependent manner by insulin at 25 nM with half maximal inhibition at 50 nM insulin. In the same experiment, the *Pck1* mRNA abundance started to be reduced in a dose-dependent manner by insulin at 5 nM and more. Figure 4C shows the time course of insulin-mediated suppression of *Enpp1* mRNA in HepG2 cells. Insulin treatment significantly reduced *Enpp1* expression as early as 3 h, and reduced total *Enpp1* mRNA abundance level by 60% at 16 h.

## Discussion

In this study, the mRNA expression level of *Enpp1* was examined in the liver samples of rats going through fasting and refeeding cycles, and in primary rat hepatocytes and human hepatoma cells treated with insulin and dexamethasone. The fact that the rat liver expresses *Enpp1* is consistent with the original observation that the ENPP1 antiserum recognized certain antigens in the homogenate of the mouse liver (13). The results of *in vivo* animal studies prompted us to further investigate the roles of hormones such as insulin and dexamethasone in the regulation of *Enpp1* expression as these hormones are major players in the regulation of expressions of hepatic genes for the glucose and lipid metabolism The *Enpp1* mRNA level was clearly up-regulated by dexamethasone and inhibited by insulin treatments in primary rat hepatocytes and HepG2 human hepatoma cells. In addition, the activation of the cAMP pathway also seems to attenuate the dexamethasone-induced *Enpp1* expression, demonstrating the complexity of *Enpp1* regulation.

In hepatocytes, insulin regulates the expression levels of a variety of genes that are important for the control of hepatic glucose and lipid metabolism. As the enzymatic activity of ENPP1 seems to be needed for its inhibitory effect on insulin signaling (24), the suppression of *Enpp1* mRNA abundance by insulin in primary rat hepatocytes and human hepatoma cells was expected. Insulin-reduced gene expression has been attributed to a heptanucleotide sequence, TGTTTTG, in promoter of genes such as *Pck1* and insulin-receptor substrate 2 as shown in Zhang et al. (22). After looking through the genomic sequence of human *ENPP1* gene promoter, we found one heptanucleotide. Whether this element is responsible for the insulin-mediated suppression of *Enpp1* gene expression remains to be determined. This insulin-reduced *Enpp1* expression may facilitate the transduction of insulin signals in hepatocytes. Indeed, when a recombinant adenovirus expressing a short hairpin RNA against mouse ENPP1 to reduce its expression was introduced to *db/db* mice, the treatment successfully reduced plasma glucose levels and improved the oral glucose tolerance test, suggesting the improvement of insulin sensitivity in those animals (25).

The inhibitory effect of insulin on *Enpp1* expression is not as robust as that on *Pck1* expression as shown in this study. This is especially obvious when the insulin-reduced expressions of both of them are compared in the presence of other hormonal and signal transduction pathways (Figures 2 and 3). The presence of insulin completely blunted dexamethasone-induced *Pck1* expression, but not *Enpp1* expression. Interestingly, the dexamethasone-induced *Enpp1* expression is disturbed when the protein kinase A pathway is activated, a result of increased intracellular cAMP level. As shown in Figure 3B, the treatment of 20 nM 8-Br-cAMP alone did not affect the *Enpp1* in hepatocytes. However, the presence of 20 nM 8-Br-cAMP prevented the dexamethasone-induced *Enpp1* expression in the same experiment. All these results indicated that the hepatic expression of *Enpp1* can be affected positively and negatively by multiple hormonal pathways. The expression of *Enpp1* in response to the cycle of fasting and refeeding probably is the combined effect of multiple hormonal pathways. The underlying regulatory mechanism of *Enpp1* expression in hepatocytes deserves further investigation, especially in the context of metabolic diseases such as obesity and diabetes.

Another interesting observation is the lack of synergistic effect of cAMP and dexamethasone in the regulation of *Enpp1* expression. In fact, the synergistic effect of cAMP and dexamethasone are commonly observed in a series of insulin-regulated genes such as *Pck1* (26). However, *Enpp1* expression level was clearly not up-regulated by the co-treatment of 8-Br-cAMP and dexamethasone, as shown in Figure 3B. The presence of 8-Br-cAMP antagonized the dexamethasone effect on *Enpp1* expression, while these two synergized to induce the *Pck1* expression in the same experiment. This observation again emphasizes the complicated hormonal regulation of *Enpp1* in the context of various metabolic states including obesity and diabetes. Chronic human metabolic diseases such as obesity and type 2 diabetes are always associated with insulin resistance and profound changes of glucose and lipid metabolic homeostasis (2). As ENPP1 has been implied to interfere with insulin signaling, it is reasonable to suggest that ENPP1 or its family members such as ENPP2 (12) play roles in the pathophysiological changes associated with insulin resistance such as hyperglycemia through elevated hepatic gluconeogenesis. Our data indicated that insulin and dexamethasone, the two hormones involved in hepatic glucose and lipid metabolism, regulate the hepatic *Enpp1* gene expression. This mutual influence and regulation between insulin and ENPP1 seem to further suggest that ENPP1 might contribute to insulin resistance through the down-regulation of insulin action and promoting hepatic gluconeogenesis. These hypotheses all remain to be investigated.

It is worth noting that in one study, ENPP1 protein level was not linked with endogenous insulin level (27). In that study, the expression level of ENPP1 protein in normal individuals was compared with that in insulinoma patients who were shown to have persistently high levels of endogenous insulin. The normal individuals were further separated into healthy, insulin resistant, and healthy insulin-sensitive individuals based on the rate of insulin-stimulated glucose disposal (M value). There was no statistical difference in endogenous ENPP1 protein levels between insulinoma patients and healthy insulin-sensitive individuals.

One potential limitation of this study is that all the biopsies were likely obtained during the fasting state, a prerequisite for most surgical operations. At this state, insulin was at its lowest level in both groups, and it is difficult to demonstrate any regulatory effect of insulin on the *Enpp1* expression in either group, especially among insulin-sensitive individuals. In addition, unlike the normal individuals, the endogenous ENPP1 protein levels were similar among insulinoma patients except in one case, consistent with our hypothesis of the suppressive effect of high level of endogenous insulin on *Enpp1* expression in these patients.

In addition, *Enpp1−/−* mice demonstrate defects in bone mineralization and health (9,10). In osteosarcoma cells, basic fibroblast growth factor induces the expression level of ENPP1 (28). *Enpp1* gene expression can also be upregulated by tumor growth factor β in chondrocytes and osteoblasts, a process that can be inhibited by interleukin-1β as reviewed by Goding et al. (29). On the other hand, insulin has been considered an anabolic signal in bone development (30). Insulin-like growth factor 1 (IGF-1) signaling pathway also plays a critical role in the development of chondrocytes and functions of osteoblasts such as energy metabolism, which in turn regulates bone remodeling and skeletal physiology as reviewed by Guntur et al. (31). Interestingly, insulin signaling pathway is also observed in osteoblasts and is critical for bone remodeling and energy metabolism (32,33). As IGF-1 and insulin signaling pathways share common components (31), they may contribute to bone health through the regulation of *Enpp1* expression or activity directly or indirectly.

ENPP1 plays a role in the production of extracellular PPi, which regulates bone density. There are some interesting observations regarding aberrant bone mineral densities in diabetic patients (34). Low bone density is commonly observed in type 1 and some later stage type 2 diabetic patients while increased vascular calcification and bone mineral density is documented in type 2 diabetic patients. If we assume that this insulin-regulated ENPP1 expression exists in cells responsible for bone health, we probably can come up with a hypothesis to explain part of the bone health problems in patients with diabetes.

**Figure 5.**
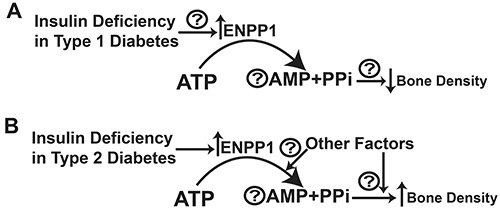
Hypothetical potential roles and remaining questions of ENPP1 in bone health changes in patients with type 1 or type 2 diabetes. *A*, In type 1 diabetes, insulin deficiency may lead to an increase of ENPP1 expression. This may cause the increase of PPi production, which may result in the reduction of bone density. *B*, In insulin resistance and type 2 diabetes, there is an increase of ENPP1 protein as well. However, this is not associated with the reduction of bone density, but rather an increase of bone density based on observations. Whether this increase of bone density is related to the change of PPi production and activities of other factors remains to be determined. The question marks indicate the steps that are worth investigating to determine the role of ENPP1 in the bone health for patients with type 1 and type 2 diabetes.

As shown in Figure 5A, the deficiency of insulin and elevation of glucocorticoids in patients with type 1 diabetes may lead to increased *Enpp1* expression, resulting in the accumulation of PPi. As an antagonist of bone development, accumulated PPi interferes with the bone developmental process in these patients, leading to reduced bone mineral density (35,36). However, this could not be used to explain the increased bone density in patient with type 2 diabetes. As it has been reviewed, elevated ENPP1 protein level and enzymatic activity are associated with insulin resistance and type 2 diabetes (29–37). This probably can be attributed to the reduced insulin action in patients with type 2 diabetes as shown in Figure 5B. In that case, an increase of PPi production and a reduction of bone density in the patients with type 2 diabetes would have been anticipated. In contrast to that, both vascular calcification and increased bone mineral density are commonly observed in some patients with type 2 diabetes, a phenomenon that could not be explained by the increase of *Enpp1* expression. Whether the increases of ENPP1 protein and bone density in insulin resistant type 2 diabetes patients is associated with changes of PPi production, and whether other factors regulate PPi production or increase of bone density without the contribution of ENPP1 remains to be determined.

Recently, mice with a mutation of myeloid-lineage leukemia gene (*Mll2*), a histone methyltransferase functioning during development, have been shown to develop hyperinsulinemia and have lowered hepatic *Enpp1* expression compared with the wildtype controls, suggesting the regulation of its expression by histone modification (38). Both histone methylation and DNA modification contribute to the gene expression pattern during development (39). It has been shown that dynamic changes of metabolites in mouse hepatocytes in response to injections of glucose and glutamate altered hepatic 5-methylcytosine hydroxylation level through ten-eleven translocation (TET) family of dioxygenases (40). The glucose injection was associated with the change of 5-methylcytosine hydroxylation profile in the promoter of mouse hepatic *Gck* promoter (40). Interestingly, that study did not test the effect of insulin injection on the TET-mediated changes. As known and shown here, insulin induces *Gck* expression. Therefore, whether enzymes such as TET1 affect insulin-reduced or dexamethasone-induced *Enpp1* expression deserves to be investigated.

In summary, we first observed the changes of *Enpp1* gene expression in the liver of rats submitted to the cycle of fasting and refeeding. This led us to observe that the expression of *Enpp1* in rat primary hepatocytes and human hepatoma cells is regulated by insulin and glucocorticoid. Dexamethasone was found to induce *Enpp1* gene expression, whereas insulin reduced it in primary hepatocytes and HepG2 cells. The implication of this observation is profound, as it may provide an important link between diabetes and abnormal bone mineral density in patients with diabetes. Further studies are needed to understand the regulatory mechanisms of *Enpp1* expression in response to changes of hormones such as insulin, and genomic modifications such as methylation, and their physiological significances.
